# Inter- and intra-observer agreement on evaluating the presence of residual glandular tissue with magnetic resonance tomography following prophylactic mastectomy

**DOI:** 10.1177/02841851211058929

**Published:** 2021-12-01

**Authors:** Märta A Skoglund, Magnus N Andersson, Annika Björkgren, Ernst Tolocka, Malin Sund, Rebecca Wiberg

**Affiliations:** 1 206100 Department of Surgical & Perioperative Sciences, Plastic surgery and Surgery, Umeå University, Umeå, Sweden; 2 211743 Department of Radiation Sciences, Oncology and Radiology, Umeå University, Umeå, Sweden; 3 Department of Integrative Medical Biology, Section of Anatomy, Umeå University, Umeå, Sweden

**Keywords:** Prophylactic mastectomy, residual glandular tissue, magnetic resonance tomography

## Abstract

**Background:**

There are no published international consensus or guideline documents regarding appropriate medical follow-up for women with hereditary increased risk of breast cancer who opt for prophylactic mastectomy. Moreover, it is not known whether breast magnetic resonance imaging (MRI) performed after a prophylactic mastectomy is a reproducible method for evaluating whether clinically relevant amounts of residual glandular tissue remains.

**Purpose:**

To evaluate the inter- and intra-observer agreement on detecting residual glandular tissue with MRI.

**Material and Methods:**

In total, 40 women previously operated with prophylactic mastectomy underwent MRI and two breast radiologists (R1 and R2) independently assessed the presence of residual glandular tissue. Inter- and intra-rater agreements were assessed using Cohen's kappa (k).

**Results:**

Residual glandular tissue was found in 69 of 248 quadrants (27.8%) and 32 of 62 breasts (51.6%) by R1 and 77 of 248 quadrants (31.1%) and 35 of 62 breasts (56.5%) by R2. The interrater agreement was observed to be moderate (k = 0.554) and the intra-rater agreement was observed to be substantial (k = 0.623).

**Conclusion:**

In conclusion, the inter-and intra-rater observer agreement in regard to detection of residual glandular tissue was not excellent, which would be desirable for a method considered reproducible enough to be used as a surveillance tool after the surgical procedure in order to ensure that there is no relevant residual glandular tissue remaining warranting further follow-up. More research is needed, as well as establishment of precise protocols, before using the method in risk assessment of remaining glandular tissue and breast cancer risk.

## Introduction

Breast cancer is the most common type of cancer among women (1), with one out of ten women in Sweden being affected during their lifetime (1). Women with pathologic variants of different breast cancer genes, such as BRCA1/2, TP53 and PTEN, have a higher lifetime risk of developing breast cancer in comparison to the overall population (2–4), and are, depending on the risk profile, recommended annual surveillance or prophylactic surgery (1). Prophylactic surgery reduces, but does not eliminate, the risk of breast cancer (5,6), with breast cancer diagnosed in approximately 1%–1.9% of all women after surgery (6). Some authors report a clear correlation between skin flap thickness and the amount of residual glandular tissue (7–10), while others do not (11); however, it is unclear whether an increased amount of residual glandular tissue translates into an increased risk of breast cancer.

There is no international consensus regarding surveillance of patients who have undergone prophylactic surgery. According to the National Comprehensive Cancer Network (NCNN) in the United States (12), women operated with risk-reducing prophylactic mastectomy should continue with annual exams of the chest/reconstructed breasts as there is still a small risk of developing breast cancer. They do not, however, specify what should be included in an annual exam, other than mammograms not being recommended in this setting.

In Germany, women undergoing bilateral prophylactic mastectomy have at least one breast magnetic resonance imaging (MRI) after the surgical procedure in order to ensure that there is no relevant residual glandular tissue remaining warranting further MRI surveillance (13). In contrast, the National Institute for Health and Care Excellence (NICE) in the United Kingdom (14) do not offer surveillance to women who have undergone prophylactic bilateral mastectomy since they consider the risk for cancer too low to justify any type of surveillance. Consistent with the NICE guidelines (14), Sweden, Norway, Denmark, and Finland do not recommend further follow-up either (1,15–17).

Due to the lack of international consensus regarding appropriate follow-up for women undergoing prophylactic surgery, the aim of the present study was to establish whether MRI, which is recommended for postoperative surveillance in some countries (13), is a reproducible method for detection of residual glandular tissue after prophylactic mastectomy.

## Material and Methods

Women with increased hereditary risk of breast cancer who had undergone prophylactic mastectomy at the Department of Plastic Surgery at Umeå University Hospital between 1997 and 2016 were invited to the study. The study was prospective in that all women who met the inclusion criteria were invited to undergo imaging surveillance with breast MRI to assess the presence of residual glandular tissue during 2017.

Inclusion criteria were women undergoing MRI postoperatively with prophylactic uni- or bilaterally mastectomized breasts reconstructed with implants or no reconstruction at all. The term “prophylactic mastectomy” is used both when there is no previous history of breast cancer in the breast, as well as in the setting of risk reducing mastectomy after a previous operation with breast-conserving surgery for cancer where information regarding increased hereditary risk motivating a risk reducing surgery has emerged later on. The term “increased hereditary risk” includes both women with pathologic variants of BRCA1/2, women with pathologic variants of other breast cancer genes, and women who after risk evaluation are considered to have an increased risk of breast cancer although no known breast cancer gene is identified. Exclusion criteria were therapeutic mastectomized breasts or breasts reconstructed with autologous tissue.

### Ethics

This study followed the principles of the Declaration of Helsinki and ethical guidelines of the Swedish Research Council. Ethical approval was obtained from the regional vetting board in Umeå (Dnr 2017-141-31M, 20170530). Since MRI of the breast is not performed routinely after prophylactic mastectomy, the investigation was initially performed for a purpose other than research. In 2016, breast cancer was diagnosed in a single patient who had undergone prophylactic mastectomy at the Department of Plastic Surgery at Umeå University Hospital. This led to the decision by the County Council of Västerbotten, as a quality assurance of hospital care, to offer all women who had been treated with this surgery to undergo a MRI of the breast to detect signs of the development of cancer, as well as establish whether the operation had been performed with enough radicality. All patients in this cohort were offered to participate in the study. All participants gave informed written consent that we could take part of the MRI results as well as review their medical records.

### Imaging analysis

All MRI examinations of the breast were performed in the prone position using a whole-body MRI scanner (Philips Medical System Achieva dStream, Nethterlands, Best) at 1.5-T main magnetic field with a dedicated seven-channel breast coil. Godateric acid (Dotarem) was used as an intravenous contrasting agent. A full MRI protocol, consisting of STIR, T1-weighted (T1W) TSE, eTHRIVE with and without intravenous contrast, and T2-weighted (T2W) TSE with intravenous contrast, was performed since the initial purpose of the imaging was a diagnostic follow-up to investigate whether the operation was performed with enough radicality or whether further surveillance was needed. All sequences were therefore used for the detection of glandular tissue. After contrast administration, dynamic sequences were acquired at five timepoints and an axial plane of acquisition was used.

Two breast radiologists (breast radiologist 1 = R1 and breast radiologist 2 = R2) analyzed the data; breast radiologist R1 started to work with breast MRI 2014 and breast radiologist R2 started to work with breast MRI 2010. They analyzed the electronic MRI image data visually, independently from each other at the same timepoint and one breast radiologist (R2) performed the analysis twice, with two months between timepoint 1 (t0) and timepoint 2 (t1). Each breast was divided into four quadrants, i.e. the inner lower, the inner upper, the outer lower, and the outer upper quadrant. Residual glandular tissue was documented as being present or not present in a preprinted clinical report form in each quadrant, in the retroareolar area if nipple-sparing mastectomy was performed and in the axilla. The residual glandular tissue was, however, not quantified.

### Statistics

Patient characteristics and frequencies of events were summarized using descriptive statistical methods with mean (SD) and percent, respectively. Inter- and intra-rater agreements with regard to detection of residual glandular tissue were assessed using Cohen's kappa (k). The strength of agreement was expressed in k values: with almost perfect agreement for values in the range of 0.81–0.99; substantial agreement for values of 0.61–0.80; moderate agreement for values of 0.41–0.60; fair agreement for values of 0.21–0.40; slight agreement for values of 0.01–0.20, and values ≤0 represented less than chance agreement (18). Data analyses were performed by SPSS version 22 (IBM Corp., Armonk, NY, USA).

## Results

### Study population

Out of an eligible population of 73 women, 29 chose not to participate and four women did not undergo MRI for different reasons: one due to claustrophobia; one due to implants containing metal; and two women due to too high body mass index (BMI) to fit into the MRI machine.

A total of 40 women met the inclusion criteria, rendering 62 breasts operated with prophylactic mastectomy reconstructed with implants (n = 60) or no reconstruction at all (n = 2) to be included in the analysis (Supplementary Fig. 1). Of these 40 women, 17 underwent bilateral prophylactic mastectomy without previous cancer, 16 underwent contralateral mastectomy, and seven underwent bilateral prophylactic mastectomy after a previous cancer operated with breast-conserving surgery. One out of 62 (1.6%) breasts was operated with simple mastectomy (SM), 35 of 62 (56.5%) breasts were operated with skin-sparing mastectomy (SSM) with an ovalar incision removing the nipple-areola complex, 24 of 62 (38.7%) breasts were operated with nipple-sparing mastectomy (NSM), and 2 of 62 (3.2%) breasts were operated with SSM with nipple transplantation.

Of the 40 women, 18 (45%) had no known mutation in breast cancer genes, 20 of 40 (50%) had a pathologic variant of BRCA1/2, and 2 of 40 (5.0%) had a pathologic variant of other breast cancer genes. The mean age at prophylactic mastectomy was 43.58 ± 10.77 years. The mean time between prophylactic mastectomy and follow-up was 7.74 ± 5.39 years, with follow-up defined as the postoperatively performed MRI. No cases of breast cancer were detected during the MRI examinations.

### Residual glandular tissue

Residual glandular tissue was found in 69 of 248 quadrants (27.8%) and 32 of 62 breasts (51.6%) by R1 and 77 of 248 quadrants (31.1%) and 35 of 62 breasts (56.5%) by R2. After NSM, residual glandular tissue was found in the retroareolar area in 13 of 24 breasts (54.2%) according to R1 and 10 of 24 breasts (41.7%) according to R2. However, looking at the breast overall, not including the retroareolar area, the amount of glandular tissue did not differ significantly between NSM and SM/SSM/SSM with nipple transplantation (RGT was found in 12 of 24 [50.0%] breasts after NSM and in 20 of 38 [52.6%] breasts after SM/SSM/SSM with nipple transplantation according to R1 and in 10 of 24 [41.7 %] breasts after NSM and in 25 of 38 [65.8%] breasts after SM/SSM/SSM with nipple transplantation according to R2, when the retroareolar area not was included in the analysis). Extramammary gland in the axilla was detected in 1 of 62 (1.6%) breasts by R1 and 2 of 62 (3.2%) breasts by R2.

**Fig. 1.**
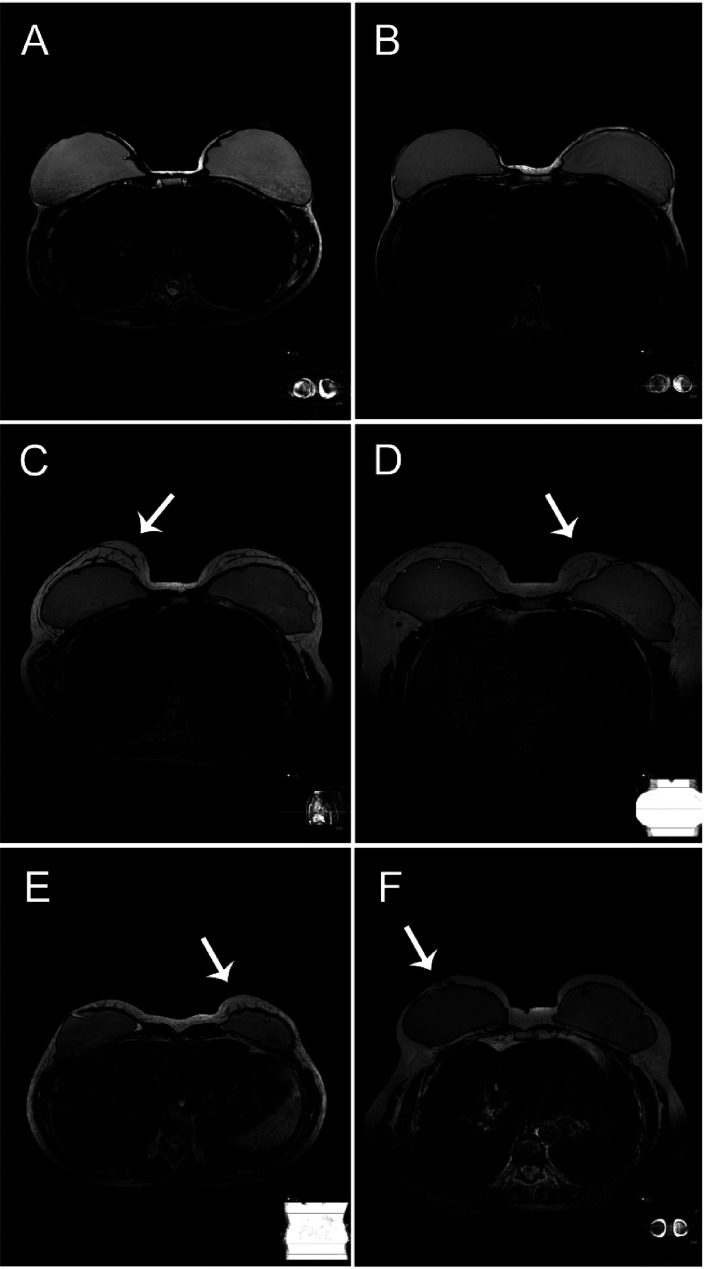
Examples on T2-weighted MRI images on women with and without residual glandular tissue postoperatively: (a, b) women with thin skin flaps without detectable residual glandular tissue; (c) woman with thick skin flaps with agreement on presence of residual glandular tissue; (d, e) women with possible residual glandular tissue where the observers disagreed whether it was residual glandular tissue, ligaments, scar tissue, artefacts, or other; (g) woman operated with nipple areolar-conserving technique with residual glandular tissue located in a retromammary area. Areas with suspected residual glandular tissue are highlighted with a white arrow. MRI, magnetic resonance imaging.

Residual glandular tissue was predominantly multifocal, 19 of 32 breasts (59.4%) had glandular tissue in more than one quadrant according to R1 and 21 of 35 breasts (60%) had glandular tissue in more than one quadrant according to R2. Examples of MRI images on women with and without residual glandular tissue postoperatively are shown in Fig. 1.

### Inter- and intra-rater agreement

Agreement between the two breasts radiologists (R1 and R2) with regard to the detection of residual glandular tissue was observed in 202 of 248 (81.5%) breast quadrants, considered a moderate agreement (k = 0.554) (Table 1). To remove the possibility that the disagreement between the two observers was due to difficulties in locating the residual glandular tissue, rather than disagreement whether residual glandular tissue was present or nor present, the interrater agreement was calculated for the superior and inferior half of the breast, the medial and lateral part of the breast, and the whole breast as well. Nevertheless, the interrater agreement was not improved by simplifying the subdivision of the breasts, thus reducing the number of measuring points (Table 1).

**Table 1.** Interrater agreement in detection of residual glandular tissue with MRI.

	Agreement between R1 and R2	Disagreement between R1 and R2	Interrater agreement (k)
Quadrants	202/248 (81.5)	46/248 (18.5)	0.554
Superior/inferior half of the breast	92/124 (74.2)	32/124 (25.8)	0.459
Medial/lateral half of the breast	91/124 (73.4)	33/124 (26.6)	0.452
Breasts	43/62 (69.4)	19/62 (30.6)	0.385

Values are given as n (%) unless otherwise indicated.

Interrater agreement in detection of residual glandular tissue with MRI was assessed using Cohen's kappa (k) by two breast radiologists (breast radiologist 1 = R1 and breast radiologist 2 = R2) in 248 breast quadrants.

MRI, magnetic resonance imaging.

Agreement for the same breast radiologist (R2) at two different timepoints (t0 and t1) with regard to the detection of residual glandular tissue was observed in 210 of 248 breast quadrants (84.7%), considered a substantial agreement (k = 0.623) (Table 2).

**Table 2.** Intra-rater agreement in detection of residual glandular tissue with MRI.

	Agreement between R2(t0) and R2(t1)	Disagreement between R2(t0) and R2(t1)	Intra-rater agreement (k)
Quadrants	210/248 (84.7)	38/248 (15.3)	0.623
Superior/inferior half of the breast	100/124 (80.6)	24/124 (19.4)	0.592
Medial/lateral half of the breast	100/124 (80.6)	24/124 (19.4)	0.591
Breasts	48/62 (77.4)	14/62 (22.6)	0.556

Values are given as n (%) unless otherwise indicated.

Intra-rater agreement in detection of residual glandular tissue with MRI were assessed using Cohen's kappa (k) by one breast radiologists (breast radiologist 2 = R2) at two different timepoints (t0 and t1) in 248 breast quadrants.

MRI, magnetic resonance imaging.

## Discussion

There is no international consensus today regarding surveillance of patients who have undergone prophylactic mastectomy. Some countries recommend postoperative MRI after the surgical procedure in order to ensure that there is no relevant residual glandular tissue remaining warranting further MRI surveillance or additional surgery (13), while other countries consider the risk for cancer too low to justify any kind of surveillance (1,14–17). Although there is enough evidence that MRI is the most suitable tool for the screening of otherwise healthy BRCA mutation carriers (19), very little has been reported concerning the use of MRI as a surveillance or diagnostic tool in patients after prophylactic mastectomy.

It is well established that a small proportion of glandular tissue often remains after any form of mastectomy, with residual glandular tissue being reported in 5%–76% of all breast specimens in histopathological reports (20,21) and in 20%–40% of all women in imaging-based studies (9,11). In line with other imaging-based studies, residual glandular tissue in the present study was found in 27.8%–31.1% of all breast quadrants and in 51.6%–56.5% of all breasts, with the residual glandular tissue being predominantly multifocal. After NSM, residual glandular tissue was found in the retroareolar area in 41.7%–54.2% of all breasts. However, looking at the breast overall, not including the retroareolar area, the amount of glandular tissue did not differ significantly between NSM versus SM/SSM/SSM with nipple transplantation. Thus, residual glandular tissue is a common postoperative finding that appears to be related to the surgical technique. When the amount of residual glandular tissue was evaluated by MRI in three subgroups of patients, bilateral prophylactic mastectomies (n = 138 breasts) with NSM performed in 78.3% of the cases, unilateral prophylactic mastectomies (n = 100 breasts) with NSM performed in 53.0% of the cases, and curative mastectomies (n = 100 breasts) with NSM performed in 46.0% of the cases, there was no significant difference between women undergoing bilateral prophylactic mastectomies and curative mastectomies, although the surgical technique differed between the groups (9). However, in another imaging-based study, where the amount of residual glandular tissue was quantified after conservative mastectomies with different surgical techniques, residual glandular tissue was found significantly more frequently after NSM than SSM (50% vs. 13%; *P* = 0.003) (11). However, the groups were not equal in size, with 69 breasts evaluated after SSM and 16 breasts evaluated after NSM. In line with the above mentioned study, a retrospective analysis of 501 breasts that had undergone postoperative breast imaging with MRI after mastectomy, showed that, excluding the areolar region, residual glandular tissue was present in 19 of 144 (13.2%) breasts after SSM and in 128 of 248 (51.6%) breasts after NSM (24). The results regarding whether the residual glandular tissue is uni- or multifocal differ in the literature. In one histopathological report (21), 206 breasts were analyzed for the presence of residual glandular tissue, with 36 samples obtained for each breast specimen. The percentage of positive samples per specimen was in the range of 1.0%–61.1%, with an average of 9.5% positive samples per specimen, diffusely found across the surface of the specimen. On the other hand, in a large imaging-based study (9), multifocal residual glandular tissue was very rare. In another imaging-based study (11), presence of residual glandular tissue was found in 19 breast quadrants when 14 breasts were analyzed, corresponding to 35.7% multifocality.

To evaluate the reproducibility of MRI, we analyzed the inter- and intra-rater agreement between two breast radiologists after prophylactic mastectomy. Our results demonstrate a moderate interrater agreement (k = 0.554) and a substantial intra-rater agreement (k = 0.623). From a surgical point of view, if the imaging is performed for the purpose of detecting residual glandular tissue warranting additional surgery, it is important to be able to locate the tissue. Therefore, the observers divided the breast into four quadrants. To remove the possibility that the disagreement was due to difficulties in locating the residual glandular tissue rather than disagreement whether glandular tissue was present or nor present, the interrater agreement was calculated for the superior and inferior half of the breast, the medial and lateral part of the breast, and the whole breast as well. Nevertheless, the interrater agreement was not improved by removing the subdivision of the breasts. Difficulties in locating the suspicious residual glandular tissue, in combination with the moderate agreement, are limiting factors that need to be addressed. According to a newly published review (22), the specificity and sensitivity of detecting residual glandular tissue with MRI is not evaluated, and neither is the clinical significance of this finding. The level of residual glandular tissue has been analyzed in some imaging-based studies (9–11,23), but often there is one radiologist performing the analysis (23), or when more than one radiologist is involved, conflicting results are discussed between the observers to find a consensus (9).

In one big imaging-based study (9), when the presence of residual glandular tissue was evaluated in 338 breasts after prophylactic mastectomy, two radiologists first evaluated the data independently and then, as a second step, discussed conflicting results between the two readers to find a consensus, with no inter- or intra-observer agreement calculated. In another big imaging-based study (23), the presence of residual glandular tissue was evaluated in 501 breasts after mastectomy, but the measurements were performed by only one radiologist. To our knowledge, there is only one study that has evaluated the reproducibility of MRI (11), where three readers reached substantial to excellent agreement with regard to the presence of residual glandular tissue in 85 breasts, which is a much stronger agreement than that demonstrated in this study. The low reproducibility showed could be due to several factors. The limited spatial resolution makes small areas with glandular tissue difficult to visualize with breast MRI, where small amounts of glandular tissue could be mistaken for ligaments, scar tissue, or artefacts (9). Additionally, the timing of the follow-up MRI could also influence the sensitivity, with areas of fat necrosis, skin thickening, seroma, and hematoma potentially complicating the assessment (24).

Even if MRI was proven to be a reproducible method for the detection of residual glandular tissue, there is still no clear correlation between an increased amount of residual glandular tissue and an increased cancer risk. Thus, the decision to implement routine surveillance has to be weighed against the potential anxiety provoked by surveillance itself.

The present study has some limitations. Although the number of measuring points was high, the cohort of included women was small. Furthermore, we had only two breast radiologists going through the material. Increasing the number of independent observers could perhaps improve the evaluation of reproducibility of the method. To further improve the quality of the paper, volumetric assessment would have been desirable, but we did not have the knowledge in our hospital to perform that kind of analysis.

In conclusion, the inter- and intra-rater observer agreement with regard to the detection of residual glandular tissue was not excellent, which would be desirable for a method considered reproducible enough to be used as a surveillance tool after the surgical procedure in order to ensure that there is no relevant residual glandular tissue remaining warranting further follow-up. Most importantly, further studies are warranted in order to assess the oncological risk associated with residual glandular tissue and to determine whether any kind of surveillance is motivated or not.

## Supplemental Material

sj-pdf-1-acr-10.1177_02841851211058929 - Supplemental material for Inter- and intra-observer agreement on evaluating the presence of residual glandular tissue with magnetic resonance tomography following prophylactic mastectomy Click here for additional data file. Supplemental material, sj-pdf-1-acr-10.1177_02841851211058929 for Inter- and intra-observer agreement on evaluating the presence of residual glandular tissue with magnetic resonance tomography following prophylactic mastectomy by Märta A Skoglund, Magnus N Andersson, Annika Björkgren, Ernst Tolocka, Malin Sund and Rebecca Wiberg in Acta Radiologica
