# Supplementary figures and images for: Inter- and intra-observer agreement on evaluating the presence of residual glandular tissue with magnetic resonance tomography following prophylactic mastectomy
Source: Acta Radiol. 2021 Dec 1;64(1):67–73. doi: 10.1177/02841851211058929 (PMC9780752; doi:10.1177/02841851211058929)

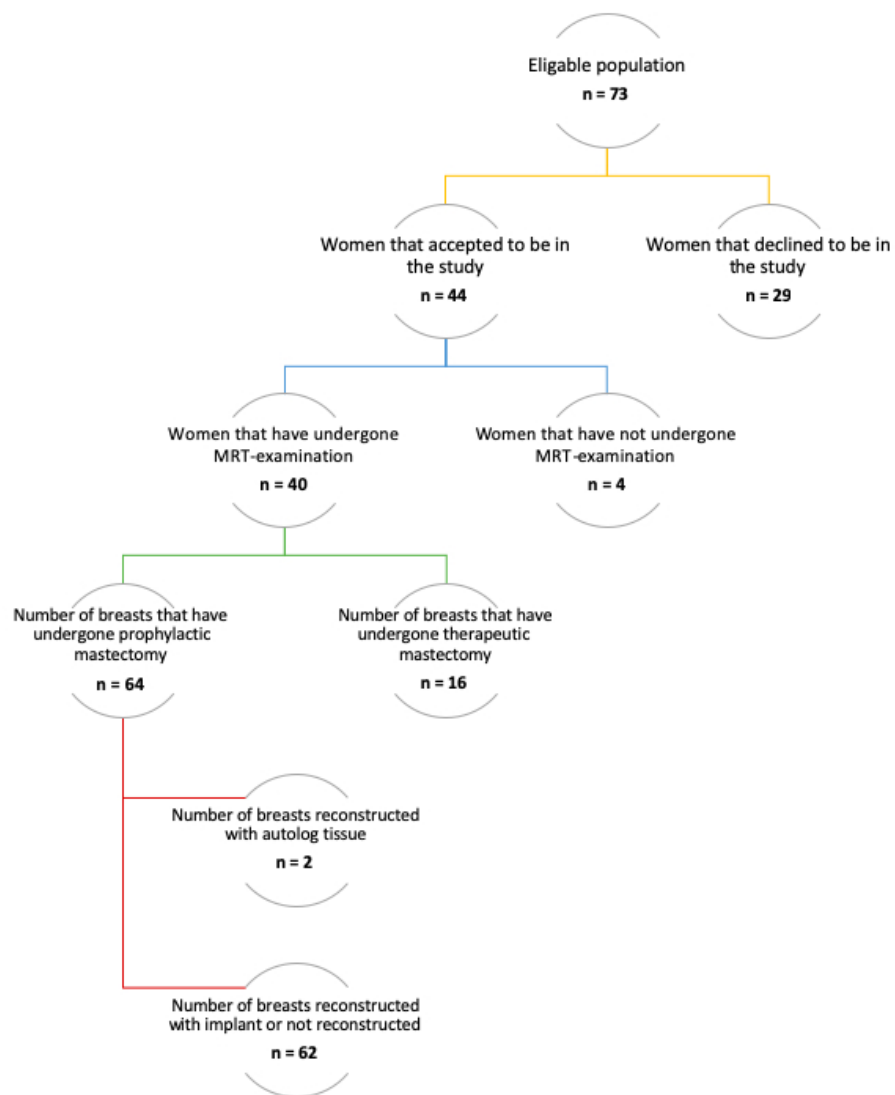

Supplementary Fig.1. Flow chart of inclusions and exclusions.

Supplement: sj-pdf-1-acr-10.1177_02841851211058929 - Supplemental material for Inter- and intra-observer agreement on evaluating the presence of residual glandular tissue with magnetic resonance tomography following prophylactic mastectomy [file sj-pdf-1-acr-10.1177_02841851211058929.pdf]
